# Cerebrospinal fluid findings in patients with psychotic symptoms—a retrospective analysis

**DOI:** 10.1038/s41598-021-86170-w

**Published:** 2021-03-30

**Authors:** Tim W. Rattay, Pascal Martin, Debora Vittore, Holger Hengel, Idil Cebi, Johannes Tünnerhoff, Maria-Ioanna Stefanou, Jonatan F. Hoffmann, Katrin von der Ehe, Johannes Klaus, Julia Vonderschmitt, Matthias L. Herrmann, Paula Bombach, Hazar Al Barazi, Lena Zeltner, Janina Richter, Klaus Hesse, Kathrin N. Eckstein, Stefan Klingberg, Dirk Wildgruber

**Affiliations:** 1 Department of Neurodegenerative Disease, Hertie-Institute for Clinical Brain Research, and Center for Neurology, University of Tübingen, Hoppe-Seyler-Str. 3, Tübingen, 72076 Germany; 2 German Center of Neurodegenerative Diseases (DZNE), Tübingen, Germany; 3 Department of Psychiatry and Psychotherapy, University of Tübingen, Tübingen, Germany; 4 Center for Rare Diseases (ZSE), Tübingen, Germany; 5 Department of Epileptology, Hertie-Institute for Clinical Brain Research, and Center for Neurology, University of Tübingen, Tübingen, Germany; 6 Department for General Neurology and Stroke, Hertie-Institute for Clinical Brain Research, and Center for Neurology, University of Tübingen, Tübingen, Germany; 7 Department of Neurology and Neuroscience, Medical Center, University of Freiburg, Breisgau, Germany

**Keywords:** Autoimmunity, Infectious diseases, Neurology, Neurological manifestations, Biomarkers, Pathogenesis, Psychology, Human behaviour

## Abstract

In current international classification systems (ICD-10, DSM5), the diagnostic criteria for psychotic disorders (e.g. schizophrenia and schizoaffective disorder) are based on symptomatic descriptions since no unambiguous biomarkers are known to date. However, when underlying causes of psychotic symptoms, like inflammation, ischemia, or tumor affecting the neural tissue can be identified, a different classification is used ("psychotic disorder with delusions due to known physiological condition" (ICD-10: F06.2) or psychosis caused by medical factors (DSM5)). While CSF analysis still is considered optional in current diagnostic guidelines for psychotic disorders, CSF biomarkers could help to identify known physiological conditions. In this retrospective, partly descriptive analysis of 144 patients with psychotic symptoms and available CSF data, we analyzed CSF examinations' significance to differentiate patients with specific etiological factors (F06.2) from patients with schizophrenia, schizotypal, delusional, and other non-mood psychotic disorders (F2). In 40.3% of all patients, at least one CSF parameter was out of the reference range. Abnormal CSF-findings were found significantly more often in patients diagnosed with F06.2 (88.2%) as compared to patients diagnosed with F2 (23.8%, *p* < 0.00001). A total of 17 cases were identified as probably caused by specific etiological factors (F06.2), of which ten cases fulfilled the criteria for a *probable* autoimmune psychosis linked to the following autoantibodies: amphiphysin, CASPR2, CV2, LGl1, NMDA, zic4, and titin. Two cases presented with anti-thyroid tissue autoantibodies. In four cases, further probable causal factors were identified: COVID-19, a frontal intracranial tumor, multiple sclerosis (n = 2), and neurosyphilis. Twenty-one cases remained with "no reliable diagnostic classification". Age at onset of psychotic symptoms differed between patients diagnosed with F2 and F06.2 (*p* = 0.014), with the latter group being older (median: 44 vs. 28 years). Various CSF parameters were analyzed in an exploratory analysis, identifying pleocytosis and oligoclonal bands (OCBs) as discriminators (F06.2 vs. F2) with a high specificity of > 96% each. No group differences were found for gender, characteristics of psychotic symptoms, substance dependency, or family history. This study emphasizes the great importance of a detailed diagnostic workup in diagnosing psychotic disorders, including CSF analysis, to detect possible underlying pathologies and improve treatment decisions.

## Introduction

Despite extensive research, the pathophysiology of schizophrenia and schizoaffective disorders has not been clarified yet^1^. In current disease classification systems (International Statistical Classification of Diseases and Related Health Problems 10(ICD-10) or Diagnostic and Statistical Manual of Mental Disorders 5(DSM-5)), psychiatric disorders are primarily classified using symptom descriptions^2,3^. However, in cases in which a specific morphologically comprehensible pathophysiological process is assumed to cause psychotic symptoms (e.g. inflammation, tumor, or ischemia), a causal attribution is made^4^.The terminology for these cases has changed in recent years. Currently, the DSM-5 defines them as "psychotic symptoms caused by a medical factor"; the ICD-10 as “psychotic disorder with delusions due to known physiological condition (F06.2)” (or organic psychosis). Exclusion diagnostics are maintained to distinguish between psychotic symptoms as part of disorders classified as F2 "Psychotic disorder with delusions due to known physiological condition" (ICD-10/DSM-5) and psychotic symptoms as part of F06.2. Exclusion diagnostics of alcohol-/drug-associated, systemic, or other brain organic causes are based on a physical examination, neuropsychological testing, routine blood testing, urine drug screening, breath/blood alcohol test, electroencephalography (EEG), and brain structural imaging (MRI) at initial manifestation^5^. Cerebrospinal fluid (CSF) analysis is considered optional. However, according to the recently updated guidelines of the German Psychiatric Association, it should be offered to patients, particularly when clinical signs indicate that psychotic symptoms might be caused by a pathological process that is associated with specific known biomarkers^6^. Psychosis was already identified as a possible immunological disease in 1930 by Lehmann-Facius amongst others, leading to the autoimmune hypothesis of schizophrenia^7–9^. In a recent consensus statement, the concept of autoimmune psychosis was updated, including the increasingly important group of autoantibody-associated autoimmune encephalitis^10–12^.

After discovering the anti N-methyl-D-aspartate receptor encephalitis in 2007, autoimmune psychosis rapidly became an important field of study^5^. Before, patients were mainly tested for antineuronal autoantibodies against intracellular antigens in the context of paraneoplastic processes. Lately, several other antineuronal antibodies against cell surface antigens (e.g., LGI1) have been associated with psychotic symptoms.

Specific etiological processes are estimated to be present in up to 25% of patients with psychotic symptoms^13^. Thus, the identification of specific underlying pathophysiologies is crucial since this may allow for more specific treatment and therefore better outcomes. Currently, immunomodulatory treatments in autoimmune psychosis^10^ or multiple sclerosis^14^, search and removal of extracranial tumors^15,16^, vitamin substitution^17^, or antibacterial treatment e.g. in syphilis^18,19^ are considered complementary to therapies for psychotic symptoms.

In this publication, we will use a descriptive approach in our nomenclature:

1. Psychotic symptoms as part of F2 "Schizophrenia, schizotypal, delusional, and other non-mood psychotic disorders" according to ICD-10 (abbr.: F2)
2. Psychotic symptoms as part of F1 "Mental and behavioral disorders due to psychoactive substance use"/F3 "Mood [affective] disorders", F4 "Anxiety, dissociative, stress-related, somatoform and other nonpsychotic mental disorders", F5 "Behavioral syndromes associated with physiological disturbances and physical factors", and F6 "Disorders of adult personality and behavior" in ICD-10 (abbr.: F1/F3-F6)
3. Psychotic symptoms as part of F06.2 "Psychotic disorder with delusions due to known physiological condition" in ICD-10 (abbr.: F06.2)
4. "No reliable classification"

In this retrospective analysis, we aimed to assess the significance of CSF examinations to differentiate psychotic disorder with delusions due to known physiological condition (label used: F06.2) from schizophrenia, schizotypal, delusional, and other non-mood psychotic disorders (label used: F2) in patients with psychotic symptoms. We hypothesized that the rate of abnormal CSF findings would be higher in patients diagnosed with F06.2 than in patients diagnosed with F2. Later age of onset is suggested to be a red flag for F06.2^20^; therefore, our secondary aim was to confirm F06.2 patients being older at disease onset than F2 patients. We aimed to identify the CSF parameters with the highest discriminatory power for F06.2 vs. F2 in a further exploratory approach. We also analyzed demographics, psychopathological symptoms, comorbidities, and family history of the cohort.

## Materials and methods

### Study description

**Figure 1:**
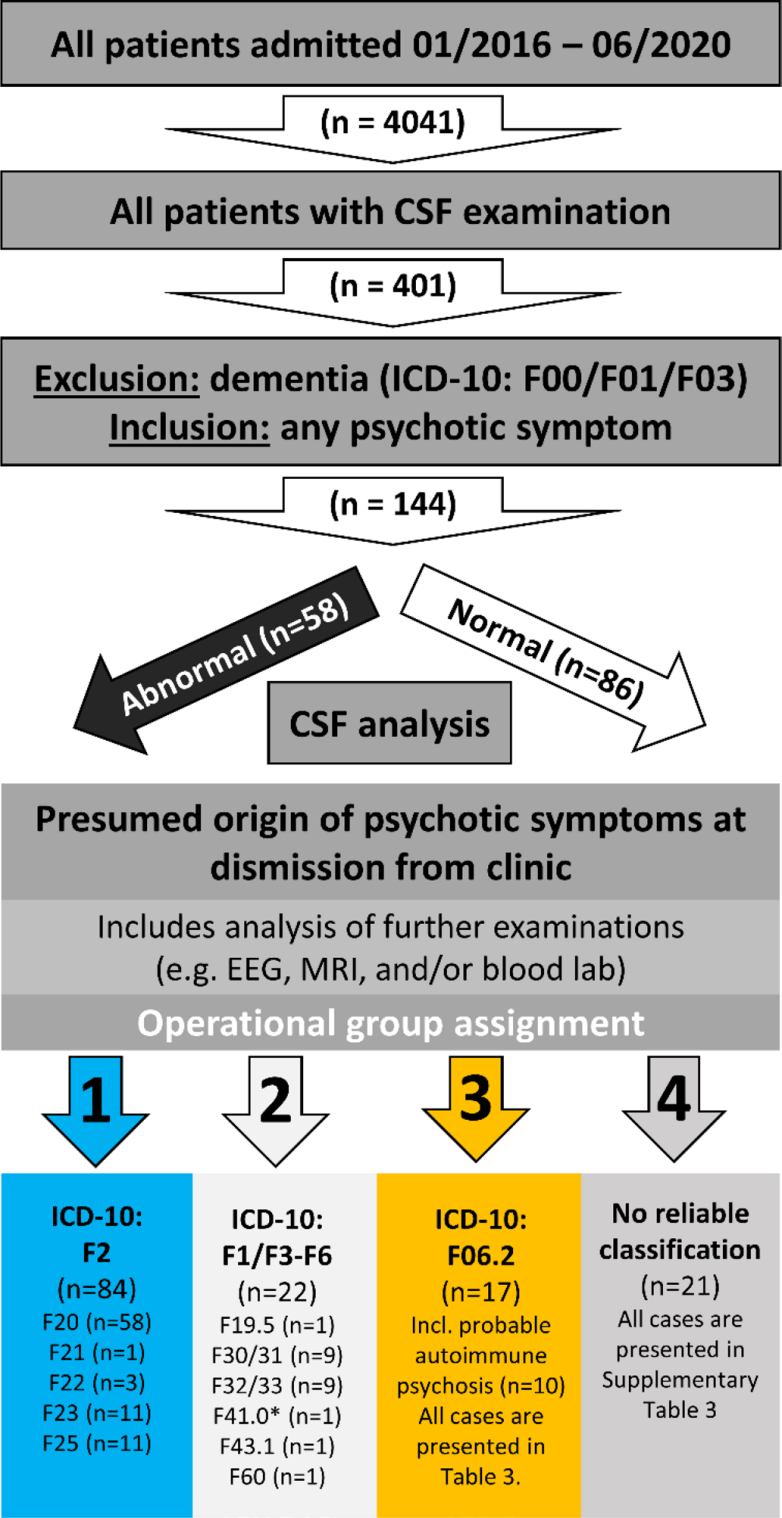
Study diagram. The study diagram shows the workflow of this retrospective analysis of n = 4041 psychiatric inpatient cases. After exclusion of cases with discharge diagnosis of dementia n = 144 cases with any psychotic symptoms and available CSF analysis were identified. These cases were dichotomized into abnormal vs. normal CSF results (for details, see Fig. 2), based on available CSF results. All cases were analyzed in detail and then operationally assigned to one of four groups, taking further examination results into account. All F2 cases constitute the first group (n = 84). The second group consists of other psychiatric disorders with psychotic symptoms (F1/F3-F6) with the exact ICD-10 diagnosis named below. (*) The F41.0 case presented with an additional Attenuated Psychosis Syndrome (as described in DSM5) with hallucinations. The F06.2 group includes the predefined *probable* autoimmune psychosis (criteria according to Pollak^10^, n = 10) and seven additional cases. In 21 cases, the abnormal findings (CSF and furthers) were not sufficient to explain the psychotic symptoms; they were categorized as "no reliable classification" due to lack of clear predefined criteria.

This study is a retrospective monocentric (Department of Psychiatry and Psychotherapy, University Hospital Tübingen, Germany) analysis of a cohort of 144 psychotic inpatients with CSF analysis, between 01/2016 and 06/2020 (42 months). For the patients recruiting scheme, see Fig. 1.

### Patient cohort

All patients admitted to any of the four acute admission wards or two wards specialized in the psychotherapy of psychotic disorders (n = 4041) were screened for CSF examination lab data. From this cohort of all CSF analyses (n = 401), patients with a discharge diagnosis of dementia (ICD-10: F00, F01, or F03) were excluded. The remaining patients were screened for any psychotic symptom (hallucinations (any modality) and delusion (any modality)) in their history or admission report (including third-party medical history); this yielded n = 144 for further in-depth analysis.

### Psychiatric and neurological assessment

Patients were assessed during their hospitalization, including a neurological examination. Psychiatric symptoms were assessed and documented by the attending physician as well as a resident physician or clinical psychologist following ICD-10 diagnostic guidelines. Additional information from previous hospital admissions was acquired, particularly regarding substance abuse/dependency, previous neurological diseases, and family history. We evaluated the plausibility for drug-induced psychosis with regard to the specific substance. For alcohol, cannabis, and cocaine, psychotic symptoms usually subside within one to four weeks after substance withdrawal^21–23^. In contrast, for hallucinogens (e.g. LSD, Psilocybin) and stimulants (e.g. Amphetamine, Methamphetamine), psychotic symptoms usually persist for up to one month and rarely up to three months or longer despite ongoing abstinence^23–25^. The necessary abstinence periods were chosen depending on the substance used (up to six months) and a diagnosis of F20 was assigned when psychotic symptoms persisted for an additional four weeks as required by the ICD-10.

### Patient sampling, including OCBs and autoantibody screening

Patients' samples (blood and CSF as paired samples) were routinely analyzed in the Institute for Clinical Chemistry and Pathobiochemistry; neurodegenerative markers and oligoclonal bands (OCBs) were analyzed in the Clinical and Chemical Laboratory "CSF laboratory", Center for Neurology, University Hospital Tübingen. OCBs were classified as types I-IV, according to the 2005 consensus statement^26^. For antibody screening, the commercially available IIFT kit, including hippocampus and cerebellum (FA 111 m-###-3) was used; for antibody differentiation, we utilized the IIFT Biochip Mosaic (FA 112d-###-6) and the anti-neuronal blot (DL 1111–1601-7 G) (all kits from EUROIMMUN, Lübeck, Germany).

### Neurodegenerative markers

The NF-LIGHT ASSAY ELISA was performed as previously published^27^. Age-dependent upper normal CSF values, as published by Yilmaz were used (Supplementary Table 1)^28^. CSF Abeta_1–42_, total tau, and phospho-tau levels were determined using commercially available ELISA kits (Fujirebio NV, former Innogenetics NV, Ghent, Belgium), cutoffs are specified in Fig. 1.

### Operationalized group assignment

**Figure 2:**
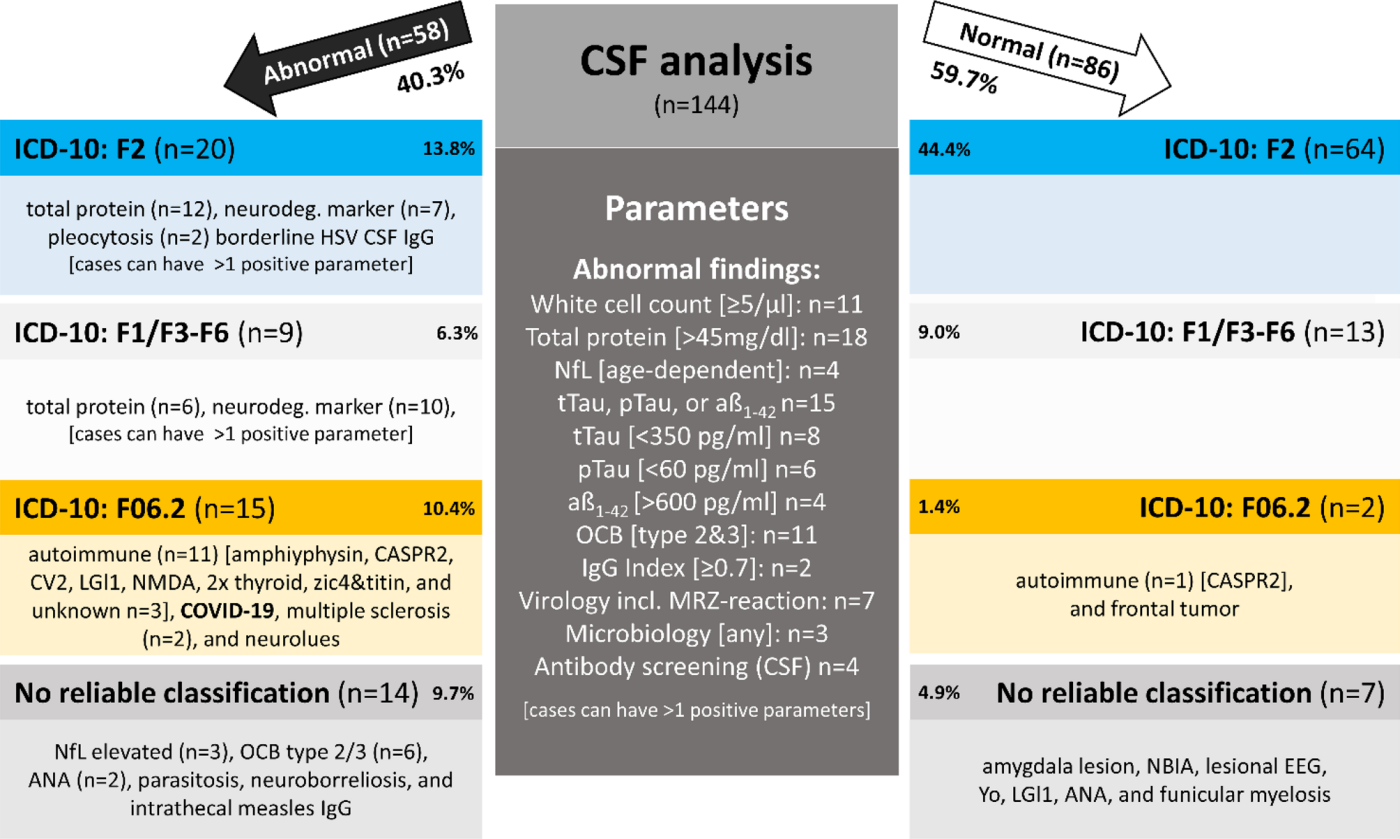
CSF analysis. The dichotomized categorization into abnormal (left) vs. normal (right) CSF was made according to the middle dark grey box's criteria. Cases can have more than one positive parameter; therefore, the sum of all findings does not add up to 100% of cases. The reported proportions are percentages of the entire cohort (n = 144). Age-dependent CSF NfL cutoffs were used according to Yilmaz^28^ (see also Supplementary Table 1). All cases were then analyzed using further examination results described in the operationalized group assignment procedure (see Fig. 1 for details).

As an operationalized group assignment, a dichotomous categorization into normal and abnormal CSF was done (Fig. 2 specifies each CSF parameters' cutoff value).

A second group allocation was based on all available clinical information, including ICD-10 diagnostic criteria, MRI and EEG findings, clinical examination, laboratory results (including CSF parameter, serum-autoantibody screening), and patients' histories. In some cases, the discharge diagnosis was adapted according to examination results to allocate the patients into four groups (see Fig. 1): (1) F2 cases, (2) F1/F3-F6 cases with psychotic symptoms, including cases with coincidental CSF abnormalities presumed unrelated to psychotic symptoms, and (3) F06.2 consisting of two subgroups: (3a) *probable* autoimmune psychosis as suggested by Pollak^10^ and due to a lack of diagnostic criteria for symptomatic non-autoimmune psychosis, (3b) patients with psychotic symptoms and a presumed causal origin. (4) Cases fulfilling criteria for *possible* autoimmune psychosis (according to Pollak) as well as cases that were not sufficiently explained with the examination results available were assigned to the "no reliable classification" category.

### Ethics approval

This study was carried out according to the Helsinki Declaration and approved by the Institutional Review Board of the University of Tübingen, reference number (291/2020BO2). According to German law and the Institutional Review Board, there was no necessity to retroactively obtain informed consent due to the importance of this study subject and the retrospective study design.

### Statistics

SPSS 26 (IBM, Armonk, NY) was used. The Shapiro–Wilk Test tested Gaussian distribution due to 3 < n < 3000. For the demographic data analysis, all variables were tested using the Mann–Whitney-U-test (being all non-Gaussian distributed variables), and the chi-square test analyzed gender as nominal values using a two-sided exact p-value. For group comparisons of the four disease classifications (F2, F1/F3-F6, F06.2, and "no reliable classification"), the Kruskal–Wallis-test was used with Dunn-Bonferroni-testing as posthoc analysis. Bonferroni correction was applied to correct for multiple testing for the two main hypotheses (prevalence of abnormal CSF findings and age at onset) presented in Table 1, including corrected *p*-values^29^.

**Table 1:** Prevalence of abnormal CSF findings and age at onset of psychotic symptoms.

Group	Any abnormal CSF finding (%)	Age at onset (years)
CSF normal (n = 86)	0	29.5 (14) [0–76]
CSF abnormal (n = 58)	100	30.5 (31) [15–73])
*p*–values (CSF normal vs. abnormal)	n.t	0.22
F2 (n = 84)	23.8	28 (15) [0–65]
F1/F3-F6 (n = 22)	40.9	27.5 (35) [15–73]
F06.2 (n = 17)	88.2	44 (24) [18–66]
*Probable* autoimmune psychosis^10^ (n = 10)	90.0	43 (33) [18–66]
Further cases (n = 7)	85.7	44 (10) [28–61]
"No reliable classification" (n = 21)	66.7	33 (26) [20–76]
*p*-values (F2 vs. F06.2)	< 0.00001	0.014

Any abnormal CSF finding is presented as prevalence percentage and being a nominal variable tested by chi-square test. *Age at onset* is presented as median (interquartile range) [range (minimum–maximum value)] tested by the Shapiro–Wilk test being a non-Gaussian-distributed variable. Only corrected p-values (two tests, Bonferroni) are reported. For group comparisons of age at onset, the Kruskal–Wallis-test was used. The posthoc analysis is presented in the results section.

Abbreviation: n.t. = not tested.

All further data analyses were exploratory and no further correction for multiple testing was applied. Bivariate correlations were analyzed using Pearson's coefficient or Spearman's rank correlation coefficient, respectively.

## Results

### CSF abnormalities

We were able to confirm our primary hypothesis of a significantly higher frequency of abnormal CSF findings in F06.2 compared with F2 with *p* < 0.00001 (Table 1). Of the 144 CSF analyses, 40.3% had at least one abnormal parameter (as specified in Fig. 2, Table 2, and the Methods Section). The most common abnormal CSF finding was an increased total protein level [> 45 mg/dl] in n = 26, followed by pleocytosis [≥ 5/µl] in n = 11 as well as positive OCBs (type III and IV) in n = 11 cases (further abnormal findings see Fig. 2). According to the operationalized group assignment, the 58 abnormal CSF examinations were divided into four groups, resulting in 15 F06.2, 14 with "no reliable classification", 20 F2, and nine F1/F3-F6 cases. Abnormal CSF occurrence, according to the different groups, is specified in Table 1 and the chapters below. Total protein in CSF did not correlate with age r_s_ (144) = 0.72, p = 0.388.

**Table 2:** Cerebrospinal fluid (CSF) findings.

Group	Cell count: white blood cells [/µl] (n = 144)	Cell count: red blood cells [1000/µl] (n = 144)	Total protein [mg/dl] (n = 144)	Quotient CSF/serum albumin (n = 125)	Quotient CSF/serum IgG (n = 125)	Oligoclonal bands (n = 99)	Total-tau [pg/ml] (n = 61)	Phospho-tau [pg/ml] (n = 59)	Abeta _1–42_ [pg/ml] (n = 61)	Neurofilament light chain (NfL) [pg/ml] (n = 42)
F2 (n = 84)	1.83 ± 1.3 [0–6]	0.15 ± 0.50 [0–3]	35 ± 11 [13–72]	5.9 ± 2.0 [1.9–11.5]	2.7 ± 1.0 [0.8–5.8]	type I: n = 36, type IV: n = 9	221 ± 102 [37–453] n = 35	39 ± 17 [15–73] n = 34	984 ± 242 [302–1417] n = 35	358 ± 176 [127–828] n = 26
F1/F3-F6 (n = 22)	1.52 ± 1.0 [0–4]	0.32 ± 1.5 [0–7]	39 ± 14 [22–75]	6.5 ± 2.8 [2.2–12.3]	3.0 ± 1.2 [0.8–5.4]	type I: n = 6, type IV: n = 3	282 ± 117[67–452] n = 10	44 ± 14[15–56] n = 9	805 ± 158 [594–1120] n = 10	375 ± 117 [288–508] n = 3
F06.2 (n = 17)	6.65 ± 10.1 [0–42]	0.50 ± 1.6 [0–6]	36 ± 14 [16–66]	6.9 ± 2.9 [2.9–13.2]	3.8 ± 2.5 [1.2–9.4]	type I: n = 4, type II: n = 4, type III: n = 1, type IV: n = 1	240 ± 99.5 [153–421] n = 6	42 ± 16 [30–64] n = 6	794 ± 236 [586–1166] n = 6	799 ± 752 [236–2080] n = 6
"No reliable classification" (n = 21)	1.48 ± 0.98 [0–3]	0 ± 0 [0–0]	36 ± 14 [19–82]	5.9 ± 2.75 [2.5–14.1]	3.1 ± 2.0 [1.3–10.2]	type I: n = 2, type II: n = 4, type III: n = 2, type IV: n = 4	231 ± 67 [129–347] n = 10	40 ± 12 [24–56] n = 10	994 ± 244 [636–1392] n = 10	764 ± 630 [321–1782] n = 7
*p*–values (F2 vs. F06.2)	**0.008**	0.68	0.76	0.58	0.33	** < 0.00001**	0.61	0.75	0.074	0.33
Gaussian variable	No	No	No	No	No	Nominal	Yes	Yes	Yes	No

All values are reported as mean ± standard deviation, range [minimum–maximum]. All variables were non-Gaussian variables and therefore tested using the Mann–Whitney-U-Test; two-sided significance level was used. OCB group differences were analyzed using Pearson's chi-square test. This data analysis was exploratory, no correction for multiple testing was applied. Bold items are below an alpha of 5%. Abbreviations: CSF: cerebrospinal fluid, OCBs: oligoclonal bands.

### Cohort

We were also able to confirm our secondary hypothesis of a significantly (p = 0.014) higher age at onset in F06.2 than F2 patients (44 vs. 28 years) (Table 1). Exploratory analysis revealed no differences in the normal vs. abnormal CSF group with regard to age (*p* = 0.11) or gender. There was a group difference regarding the presumed underlying pathology when comparing F06.2 with F2 for age at examination (*p* = 0.036—compare Table 3). There were no significant group differences in current substance dependency or family history for any psychotic disorder. The lack of group differences applies to both the distinction of CSF normal vs. abnormal and for the presumed underlying disease pathology (Table 3).

**Table 3:** Demographics, psychopathological symptoms, comorbidities & family history of the cohort.

Group	Age [years]	Gender		Hallucination (any)	Acoustic	Visual	Tactile	Delusion (any)	Disorganized thinking and speech	Catatonic symptoms	Negative symptoms	Depressive symptoms	Mnestic or orientation difficulties	Comorbidity: substance dependency (previous) [%]	Comorbidity: substance dependency (previous) [%]	Positive family history for F2/F30/F31 disorder [%]	Positive family history, any psychiatric disorder [%]
		♀	♂														
CSF normal (n = 86)	34 (21) [19–77]	37	49	52.3	33.7	20.9	5.8	84.9	79.1	11.6	45.3	60.5	48.8	44.2	50.0	29.1	40.7
CSF abnormal (n = 58)	43 (34) [19-79]	19	39	55.2	37.9	27.6	5.2	77.6	74.1	5.2	53.4	70.7	60.3	32.8	43.1	17.2	39.7
*p*–values (CSF normal vs. abnormal)	0.11	0.65		0.74	0.60	0.36	0.87	0.26	0.49	0.19	0.34	0.21	0.18	0.17	0.42	0.11	0.90
F2 (n = 84)	33.5 (21) [19–67]	32	52	50.0	36.9	17.9	6.0	86.9	78.6	14.3	47.6	61.9	47.6	45.2	50.0	27.4	36.9
F1/F3-F6 (n = 22)	42.5 (46) [20–76]	8	14	59.1	27.3	31.8	0	63.6	63.6	4.5	36.4	68.2	59.1	36.4	36.4	18.2	45.5
F06.2 (n = 17)	51 (26) [22–67]	8	9	47.1	23.5	35.3	11.8	88.2	88.2	0	47.1	58.8	64.7	23.5	41.2	23.5	47.1
*Probable * Autoimmune psychosis^10^ (n = 10)	45 (33) [22–67]	5	5	40.0	20.0	20.0	10.0	80.0	90.0	0	70.0	70.0	70.0	20.0	40.0	20.0	40.0
Further cases (n = 7)	52 (14) [28–62]	3	5	57.1	28.6	57.1	14.3	100	85.7	0	14.3	42.9	57.1	28.6	42.9	28.6	57.1
"No reliable classification" (n = 21)	39 (30) [20–79]	8	13	66.7	47.6	28.6	4.8	76.2	76.2	0	66.7	76.2	61.9	33.3	52.4	19.0	42.9
*p*–values (F2 vs. F06.2)	0.036	0.49		0.83	0.29	0.11	0.39	0.88	0.36	0.10	0.81	0.97	0.20	0.10	0.51	0.74	043

Age is presented as median (interquartile range) [range (minimum–maximum value)]. Since there was no missing data for the subgroups, type of psychotic symptom and comorbidities are reported as prevalence percentage values.Age was non-Gaussian-distributed (tested by the Shapiro–Wilk test); all remaining values were tested by Pearson's chi-square. Two-sided significance values are reported.

### Clinical findings—psychotic symptoms

All psychotic symptoms (according to ICD-10 diagnostic criteria for schizophrenia) did not differ between the groups in our exploratory analysis (see Table 3).

In addition, there were no group differences between the F2 and the "no reliable classification" group neither for psychotic symptoms nor any comorbidity or positive family history. Severity of depressive symptoms (rated 1–3 according to individual patients history as expert opinion by the study team) was not different at a group level (*p* = 0.09) when comparing "no reliable classification" (median: 1 ± IQR: 2) [range: 0–3]) vs. F2 (1 ± 1 [0–3]) as well as hallucination modalities (number of different hallucination modalities; *p* = 0.172) for "no reliable classification" (1 ± 1 [0–2]) vs. F2 (0 ± 1 [0–3]) as well as (depression *p* = 0.95; hallucination *p* = 0.61) F06.2 (depression strength (1 ± 2 [0–2]); hallucination modalities (0 ± 2 [0–2])) vs. F2.

### F06.2

This group is composed of two subgroups: a) *probable* autoimmune psychosis (according to Pollak^10^) (n = 10) and b) psychosis of probable other etiology (n = 8). Since we did not find well-operationalized criteria for non-autoimmune symptomatic psychosis in the literature, we tried to apply analogous criteria and counted six cases, which presumably have an identifiable underlying pathology (non-bold cases in Table 4). CSF examination (Fig. 2) revealed five cases with abnormalities: anti-thyroid antibodies (anti-TG, TRAK, anti-TPO, and anti-TRAb) (#16&#77), COVID-19 (#111), neurosyphilis (#30), and twice OCBs type II and typical white matter lesions on brain MRI, that were diagnosed as multiple sclerosis (#49&51). In one case, the CSF examination was unremarkable, but brain MRI revealed frontal located intracranial tumor suspected to be a fibrotic dysplasia infiltrating frontal and sigmoid sinuses (#32). Two anti-thyroid autoantibody-associated cases were not classified as probable autoimmune because Pollak et al.^10^ stated that the significance of thyroid antibodies for autoimmune psychosis is still unknown. One case (#16—SREAT) had highly elevated TPO-antibodies and TRAK-antibodies in serum; the other case (#77) is mentioned above in the positive CSF findings.

**Table 4:** All cases with diagnosis of F06.2

ID	Diagnosis	♀/♂	Age (years)	Duration (years)	Clinical findings/lab results/comments
**12**	**LGl1-encephalitis**	♂	67	1	Subacute short-term > long-term memory impairment with disorganized thinking and speech. LGl1 antibodies in serum and CSF positive. Cognitive improvement after IVIG. Until 12/2019, five cycles of immunoglobulins (1 g/kg body weight over two days each) with continuous improvement
16	SREAT	♂	62	1	Subacute short-term > long-term memory impairment with disorganized thinking and speech. SREAT (steroid-responsive encephalopathy associated with autoimmune thyroiditis). Highly elevated TPO-antibodies and TRAK-antibodies, improvement under steroid therapy
**26**	**Schizoaffective disorder with CSF pleocytosis and positive OCBs**	**♂**	56	2	Two-year history of psychotic symptoms. CSF examination revealed pleocytosis with leukocytes 8/µl, elevated total protein, and OCBs type II (specific intrathecal IgG production). EEG unremarkable, brain MRI with unspecific white matter lesions, no novel lesions in 1.5 years follow up, no contrast agent uptaking lesions
30	Neurosyphilis	♂	55	11	Chronic progredient neurosyphilis since 2007 with psychotic symptoms (among others acoustic hallucinations) and previous dependent and compulsive behavior. Initially (2007) treated with iv antibiotics, stable phase until 2011 with reactivation of the neurosyphilis. In 2019 another reactivation with improvement under intravenous penicillin G 3 × 10 million IU for 14 days
32	Frontal intracranial tumor	♀	52	7	Brain MRI revealed an intracranial frontal located tumor (suspected fibrotic dysplasia) with infiltrations of the frontal and sigmoid sinuses. Slow tumor growth over the past four years with progredient disorganized thinking and speech and paranoia, including persecutory delusions. Inconclusive CSF and blood tests with anti-neuronal autoantibodies, including testing for unspecific bindings in mouse, rat, and primate brain sections
**33**	**CASPR2-associated psychosis with ovarian teratoma (case study published**^15^)	♀	53	1	Depressive episode (approx. four weeks) with suicidal thoughts and atypical paranoia (including persecutory and guilt delusions). Ego-disturbance as well as disorganized thinking and speech. Did not improve significantly after antidepressive treatment. Blood examination revealed Caspr2-autoantibodies in serum,whole-body staging an ovarian teratoma, which was surgically removed. Psychotic symptoms that initially did not respond well to antipsychotic treatment quickly resolved after teratoma removal and unchanged continuous treatment with olanzapine 20 mg/d
**39**	**CV2-AB mediated psychosis with cerebellar ataxia**	♀	51	2	Two-year history with psychotic and affective symptoms, saccadic pursuit, atactic gait and stance as well as knee-chin testing with atactic features. Cerebellar atrophy in brain MRI. Positive CV2-blot in CSF and serum. Self-discharged before treatment attempt
49	Acute polymorphic psychotic disorder with intrathecal IgG production as an unusual manifestation of multiple sclerosis	♂	41	0	Acute polymorphic psychotic disorder with disorganized thinking and speech as well as paranoia (mostly persecutory delusions). OCB positive (type III), CSF IgG index increased, brain MRI in accordance with multiple sclerosis (white matter lesions in typical locations including the corpus callosum). No infectious or autoantibody-mediated positive lab results in CSF or serum. Methylprednisolone treatment (5 days, 1 g IV each day) initially, then prednisolone orally tapered beginning with 60 mg over eight weeks) with a reasonable reduction of psychotic symptoms with concurrant reduction of haloperidol treatment
51	Psychotic symptoms in multiple sclerosis	♀	41	0	Known > 13 years history of relapsing–remitting multiple sclerosis, initially treated with IV methylprednisolone (5 days, 1 g each), then natalizumab, then fingolimod. No treatment at admission. Approx. onset of psychotic symptoms > 2 years with disorganized thinking and speech, paranoia (mostly persecutory and control delusions), thought broadcasting, and thought insertion. Brain MRI revealed no contrast-agent absorbing lesions; white matter changes pattern in accordance with multiple sclerosis. OCBs type II with specific intrathecal IgG production
77	Acute psychotic symptoms in thyroid mediated antibody-positive encephalitis with anti-TG, TRAK, anti-TPO, and anti-TRAb AB	♀	28	0	Acute onset (two to three weeks) with disorganized thinking and speech, paranoia (persecutory delusions), acoustic hallucinations and thought insertion. CSF examination revealed multiple positive intrathecally identified thyroid targeted antibodies, including anti-TG, TRAK, anti-TPO, and anti-TRAb antibodies. Recently diagnosed graves disease, no continuous adherence to the prescribed carbimazole. Treated with carbimazole after admission without improvement; methylprednisolone treatment (5 days, 1 g IV each day) lead to quick improvement of psychotic symptoms (with continuous olanzapine treatment (10 mg/d))
**98**	**Acute polymorphic psychotic disorder, intrathecal IgG production and serum anti-amphiphysin AB**	♂	22	1	Acute onset of psychotic symptoms with optic and acoustic hallucinations (voices calling his name) and derealization. Brain MRI without contrast-agent absorbing lesions or any specific white matter lesions. CSF examination revealed OCB type II (specific intrathecal IgG production). Serum with anti-amphiphysin antibodies, not found in CSF. Existing absence epilepsy with sharp waves and poly spikes in EEG recordings. Manifest vitamin-D deficiency
111	Acute polymorphic psychotic disorder associated with COVID-19 infection	**♂**	52	0	Admission with acute onset of psychotic symptoms combined with respiratory symptoms (coughing and dyspnea) and hyposmia. CSF examination revealed pleocytosis and elevated total protein. Further microbiological and virological findings unremarkable. Brain MRI was unremarkable. TIA symptoms with temporary quadrant vision loss during hospitalization; new brain MRI and specifically DWI sequences unremarkable. A possible contribution of COVID-19 disease to manifestation of florid psychotic symptoms is probable – close time-related coincidence of psychotic exacerbation and PCR detection of the SARS-CoV2 virus in a throat swab
**116**	**Psychosis with CASPR2-AB in serum**	♀	39	2	Two-year history of psychotic symptoms with preceding paranoia for further two years and social isolation for about eight years prior. EEG with intermittent theta activity. MRI was non-remarkable. No tumor was found despite detailed imaging and clinical examinations
**118**	**Psychotic symptoms with CSF pleocytosis**	♂	25	2	Two-year history of psychotic symptoms. CSF pleocytosis with additional parameters unremarkable. Unremarkable brain MRI and EEG
**131**	**Acute polymorphic psychosis with CSF pleocytosis**	♀	62	0	Four-year history of social isolation. Acute onset of psychotic symptoms (about ½ year). CSF examination revealed CSF pleocytosis. Brain MRI with global unspecific atrophy supra- and infratentorial, further MRI findings unremarkable including contrast-agent and unremarkable EEG. No tumor was found despite detailed imaging and clinical examinations
**139**	**Psychosis with anti-Zic4 and anti-titin antibodies in serum**	♂	18	7	Nine-year history of psychotic symptoms. EEG examination showed intermittent theta activity. CSF examination revealed pleocytosis and serum anti-neuronal antibodies against Zic4 and titin. Brain MRI was unremarkable (no contrast-agent)
**142**	**Anti-NMDA encephalitis**	♀	31	0	Acute onset of progressive aphasic syndrome with psychotic symptoms over three weeks EEG with epilepsy-typical potentials in left frontal electrodes, recognized as a possible lesion. Pleocytosis (42 cells/µl) with anti-NMDA antibodies in serum and CSF positive. MRI unremarkable. Treated with IgG (initially 120 mg over four days) with clinical improvement, regime twice repeated at intervals of six weeks with further improvement. No tumor was found despite detailed imaging and clinical examinations

The cases listed in bold (n = 10) are probable autoimmune psychoses according to Pollak. The non-bold cases (n = 7) are categorized as other F06.2 cases as described in the Methods Section because an attributable and explanatory process was identified. The cases are summarized with the abnormal examination reports listed above. Abbreviations: AB: antibodies, OCB: oligoclonal bands.

### *Probable* autoimmune psychosis

A total of ten cases (6.9% of the cohort with psychotic symptoms of n = 144) were classified (according to Pollak et al.^10^) as *probable* autoimmune psychosis and are listed in Table 4, including gender, age, diseases duration, as well as a summary of the clinical findings. CSF analysis for autoantibodies (Fig. 2) revealed 33.3% of those (n = 3) to be of autoimmune origin (CV2, LGl1, NMDA). Of the remaining seven cases, serum analysis for autoantibodies led to the diagnosis of four further *probable* autoimmune psychosis cases (57.1%): anti-CASPR2 associated psychosis with ovarian teratoma (case #33, previously published by Herrmann^15^), anti-CASPR2 associated psychosis without confirmed tumor (#116), serum anti-zinc finger protein 4 (zic4) and anti-titin (#139), and anti-amphiphysin- (#98). Three cases were without serum or CSF antibodies, but CSF pleocytosis as the *probable* autoimmune psychosis defining parameter according to Pollak^10^.

The exploratory comparison of different CSF parameters with regard to their discriminatory power between F06.2 and F2 revealed pleocytosis and OCB to be the most relevant parameters (Table 2). Significant group differences (F06.2 vs. F2) were found for white blood cell count (*p* = 0.008), but no differences for total protein, albumin quotient (CSF/serum), as well as IgG quotient (CSF/serum). OCB prevalence in the F06.2 group was higher for types II and III than in F2 as tested by chi-square testing *X*^2^ (4, n = 68) = 23.974, *p* < 0.0001. There were no group differences in CSF neurodegenerative markers (total-tau, phospho-tau, aß_1-42,_ and NfL).

### "No reliable classification"

A total of 21 cases remained unresolved despite intensive diagnostics and were therefore categorized as "no reliable classification". The psychotic symptoms were found not to be sufficiently explained by the abnormal examination results, including *possible* autoimmune psychosis according to Pollak. However, the abnormal test results also did not allow classification as F2 or F1/F3-F6. All cases are listed in Supplementary Table 2, including gender, age, disease duration, and a summary of the clinical findings. Exploratory analysis of CSF parameters (Table 2) did not reveal any significant group differences compared to F2 for white blood cell count, total protein, albumin quotient (CSF/serum), IgG quotient (CSF/serum), and neurodegenerative markers. OCB prevalence in the "no reliable classification" group was higher for types II and III than in F2 as tested by chi-square testing *X*^2^ (4, n = 72) = 27.66, *p* < 0.0001, and there was a significant higher NfL level as tested by Mann–Whitney-U-test (*p* = 0.014) in the "no reliable classification" compared to F2.

## Discussion

There was a high prevalence of abnormal CSF findings (40.3% for all cases) in the study cohort. According to our primary hypothesis we observed a higher prevalence in F06.2 (88.2%) compared to F2 (23.8%). Pleocytosis seems not to be very sensitive (sensitivity of 47.1% to distinguish between F06.2 and F2) for the identified autoimmune psychosis cases despite being one of Graus' criteria^5^ for possible autoimmune encephalitis as well as Pollak's^10^ for *probable* autoimmune psychosis. In contrast, our cohort's specificity for pleocytosis (F06.2 vs. F2) was relatively high with 96.4% and only three cases (3.6%) being false-positive. CSF protein was elevated in 18.1% of cases, markedly less than a similar cohort published by Endres^7^ or half of the prevalence (40%) in n = 188 first episode schizophreniform syndromes^30^.

OCBs with specific (type II) or partially specific (type III) intrathecal IgG production, according to Freedman^26^, seem to be relatively common in patients with psychotic symptoms^30^ (Table 2), supporting the 90 years old autoimmune hypothesis of schizophrenia^9^. This hypothesis of mild encephalitis in at least subgroups of patients with psychotic symptoms, postulated e.g. by Bechter^31,32^, is supported by our findings. The sensitivity of OCBs to distinguish between F06.2 and F2 was 50% with a specificity of 100% but a false negative rate of 50% (n = 5). In our "no reliable classification" group (Supplementary Table 2 further cases with a longer-standing history of psychotic symptoms without MRI features and any motor manifestations according to their history were identified with OCBs (primarily type II and type III). All cases with OCB were categorized at least as "no reliable classification". Despite intrathecal IgG production and broad antibody search, antibodies were not specified. Most of these cases would be classified as possible autoimmune psychosis by Pollak^10^. Therefore, we propose that these cases should be reanalyzed and included in international or national networks like GENERATE (the GErman NEtwork for Research on AuToimmune Encephalitis) to unravel further anti-neuronal targets in autoimmune psychosis.

To our knowledge, the occurrence of OCBs in healthy control subjects has not yet been systematically studied; findings vary from 0% (n = 105^33^), 4% (n = 50^34^) to up to 7% (n = 41^35^). In contrast, in all four operationalized groups combined, about 11% of cases had OCB type II or III; this is almost double the occurrence rate noted in healthy controls. Endres et al.^30^ found 10% of OCB type II and III in n = 188 first episode schizophreniform syndromes. Healthy siblings of MS patients had 18–20% positive OCBs, probably vastly overestimating OCBs in healthy subjects, as there are shared genetic and environmental influences^34,36^; but not all OCB-positive cases should routinely be considered an autoimmune psychosis. OCB-negative MS cases depend on genetic variants being major determinants of CSF antibody levels^37^. We presume genetic predisposition plays a role concerning antibody levels and OCBs in psychosis.

NfL is a rather unspecific biomarker for axonal degeneration, showing increased CSF concentrations in neurodegenerative diseases^38^ and autoimmune diseases like MS^39^. Otto showed that NfL helps to differentiate F2/F3 disease from the behavioral variant of frontotemporal dementia^40^. In our cohort, NfL aids on a case level (despite the small sample size of n = 42) to discriminate F06.2 from F2 (Fig. 2) this in spite of the lack of significant group differences: NfL was elevated in LGl1 (#12) and neurosyphilis (#30) as well as in three further cases with "no reliable classification" (#1, 120 & 133). We suggest to further study NfL as a screening tool to guide diagnostics. The neurodegenerative markers total tau, phospho-tau, and abeta_1-42_ did not discriminate between F2 and F06.2 (Table 2). The largest cohort (n = 359) of neurologically and psychiatrically healthy adults was published by Yilmaz et al.^28^, in which 3.3% (n = 12) had NfL levels above the upper reference of two standard deviations. Therefore, even in healthy controls, increased NfL levels can occur, explaining some of the "no reliable classification" cases with increased NfL levels.

The occurrence of antinuclear antibodies (ANA) in healthy individuals seems to be stable throughout different age groups and ranges from 31.7% (1:40 serum dilution), 13.3% at 1:80, 5.0% at 1:160, to 3.3% at 1:320 in a published putative normal population^41^. Therefore, the high number of ANA-positive cases in our "no reliable classification" group is not surprising. ANA have been discussed to play a role in affective disorders, but studies showed mixed results (reviewed in ^42^); this also applies for schizophrenia (11% in an exemplary study^43^ for n = 28) with an extensive study of n = 368 patients and n = 283 controls showing no differences in ANA occurrence, but a prevalence of 11% of ANA positive cases in healthy controls^44^.

Case #11 was initially categorized in the "no reliable classification" group. However, the association of COVID-19 and psychotic symptoms was just recently described in larger cohorts^45,46^, leading to categorization in the F06.2 category. The patient presented with acute onset of psychotic symptoms combined with respiratory symptoms (coughing and dyspnea), hyposmia, CSF pleocytosis, and elevated total protein. There was a close coincidence of psychotic exacerbation and PCR detection of the SARS-CoV2 virus in a throat swab with simultaneous improvement of psychotic and respiratory symptoms through the course of the disease. Parra et al.^47^ report further evidence of a causal relationship of COVID-19 and psychosis beyond acting as a stress factor.

When analyzing our *probable* autoimmune psychosis cases (n = 10) with regard to psychiatric symptoms and clinical features, only the CASPR2 case^15^ had the "red flag symptom" hyperkinesia, as described by Herken and Prüss (others: seizures, catatonia, or autonomic instability)^48^. No catatonia was found in the subgroup of *probable* autoimmune psychosis. Two anti-thyroid autoantibody cases (#16 SREAT, #77) were not classified as probable autoimmune psychosis. Pollak et al.^10^ rate thyroid antibodies for autoimmune psychosis to be still of unknown significance. When disregarding the anti-thyroid antibodies, the two cases did not meet criteria for a probable autoimmune psychosis according to Pollak.

In a cohort of > 300 MRI and CT brain images in first episode psychosis, a prevalence of approximately 2% of structural brain abnormalities related to psychosis was reported^49^. Endres^30^ reports up to 65% of MRI imaging abnormalities, including e.g. white matter lesions, which are not necessarily related to psychosis. Despite our smaller sample sizes and even without systematic analysis of brain imaging in this cohort, we identified six cases (4.2%) with structural brain abnormalities (#2 funicular myelosis, #16 SREAT, #32 intracranial tumor, #49&#51 both MS and #67 amygdala) which more than doubles the previously reported prevalence related to psychosis.

In a systematic review of MS and psychosis (n = 91), most patients did not have a history of MS or psychiatric disease prior to manifestation of psychotic symptoms^50^. With two patients with psychotic symptoms possibly relating to MS, one case (= 50%) had a previous history of MS (> 13 years). In contrast, the second case (= 50%) presented with psychotic symptoms as the initial MS manifestation. Case #98 (compare Table 4) presented with acute onset of psychotic symptoms and clonal intrathecal IgG synthesis without MR suspect lesions, therefore not fulfilling the latest McDonald criteria^51^. However, we categorized this case as a monomorphic autoimmune process, a *probable* autoimmune psychosis, according to Pollak^10^.

The presence of antibodies in serum in conjunction with their lack in CSF can be a further complicating aspect in diagnosis. For example, CASPR2 is often undetected in CSF, but titer above 1:200 in serum have a high diagnostic value (discussed in^15^). We furthermore identified a small number of cases with other serum antibodies only (Supplementary Table 2), which could not be fully resolved with regard to diagnosis. When comparing the frequency of identified autoimmune psychosis, all antibodies but LGl1 (n = 2) occurred only once in our cohort. Surprisingly, only a single patient has been found to have anti-NMDAR encephalitis (#142), which is thought to be the most common antibody-mediated encephalitis and often exhibits psychiatric—including psychotic—symptoms^12^. In the Endres cohort^7^, anti-NMDAR encephalitis was also underrepresented, possibly due to a similar recruitment technique of psychotic patients in a tertiary psychiatric setting.

This study stands out for its large overall sample size, extensive file research and antibody analysis, as well as the explorative data on CSF levels of neurofilament light chain and non-autoimmune cases with psychotic symptoms in patients with "psychotic disorders with delusions due to known physiological condition (F062.)", including COVID-19. The lack of computerized automated recognition of symptoms from medical files and structured assessment of psychopathology constitutes one of its major limitations. Because of its retrospective nature and open and uncontrolled design, not all patients received the same diagnostic regime. This also precluded analysis of positive findings in healthy adults, similar to a recently published cohort^30^ emphasizing the need for comparative control groups in future studies, which was lacking in this study. For patients with OCBs with a positive MRZ-reaction or a positive IgG titer, a tissue-based assay to identify unknown antibodies would have been helpful. Drug-induced psychosis was judged on a case-by-case basis with the needed abstinence period for a F2 diagnosis chosen based on the substance(s) used (see Material&Methods); thus, there could be some diagnostic uncertainty there as well.

## Conclusion

Preventing damage to patients in our care is of utmost priority in medicine. A balance must be struck between economic costs and associated risks for patients to administer medical procedures effectively. The extraction of CSF using atraumatic lumbar punctures has a sensible benefit/risk ratio and should be offered to patients with psychotic symptoms^52^. In our cohort, 40.3% of cases had abnormal CSF findings (n = 58) with a significantly higher prevalence in the psychotic disorders with delusions due to known physiological condition (labeled: F06.2) (83.3%) than schizophrenia, schizotypal, delusional, and other non-mood psychotic disorders (labeled: F2) (23.8%) group. Of the ten cases with an autoimmune psychosis, nine were identified by CSF examinations. Later onset was found in patients diagnosed with F06.2 compared to F2 (median age difference between patient-groups: 16 years), emphasizing the importance of CSF analysis in patients with advanced age at disease onset. We were able to identify pleocytosis and OCBs to be good discriminators between F06.2 and F2 with high specificity (> 96%). As mounting evidence points to specific underlying pathophysiological processes in psychotic disorders, serum and CSF antibodies should be routinely used for diagnostics.

## Supplementary Information

Supplementary Information

## Data Availability

The data sets for this manuscript are not publicly available. No consent for open sharing has been obtained. Raw data regarding human subjects (e.g., genetic raw data, personal data) cannot be shared freely to protect the privacy of the human subjects involved in this study. Requests to access the data sets should be directed to Dr. Tim W. Rattay.

## Abbreviations

AB: Antibody
Abbr.: Abbreviated
ANA: Antinuclear antibodies
CSF: Cerebrospinal fluid
DSM-5: Diagnostic and Statistical Manual of Mental Disorders 5
EEG: Electroencephalogram
F06.2: "Psychotic disorder with delusions due to known physiological condition" according to ICD-10
F1: "Mental and behavioral disorders due to psychoactive substance use" according to ICD-10
F2: "Schizophrenia, schizotypal, delusional, and other non-mood psychotic disorders" according to ICD-10
F3: "Mood [affective] disorders" according to ICD-10
F4: "Anxiety, dissociative, stress-related, somatoform and other nonpsychotic mental disorders" according to ICD-10
F5: "Behavioral syndromes associated with physiological disturbances and physical factors" according to ICD-10
F6: "Disorders of adult personality and behavior" according to ICD-10
FNR: False-negative rate
ICD-10: International Statistical Classification of Diseases and Related Health Problems 10
MRI: Magnetic resonance imaging
MS: Multiple sclerosis
NBIA: Neurodegeneration with iron accumulation
NfL: Neurofilament light chain
OCBs: Oligoclonal bands
SREAT: Steroid-responsive encephalopathy associated with autoimmune thyroiditis
