# Supplementary material for: Cerebrospinal fluid findings in patients with psychotic symptoms—a retrospective analysis
Source: Sci Rep. 2021 Mar 30;11:7169. doi: 10.1038/s41598-021-86170-w (PMC8010098; doi:10.1038/s41598-021-86170-w)
Supplement: Supplementary file 1 — Supplementary Information [file 41598_2021_86170_MOESM1_ESM.docx]

**Cerebrospinal fluid findings in patients with psychotic symptoms – a retrospective analysis**

Tim W. Rattay^1,2,3,#^, Pascal Martin^3,4^, Debora Vittore^3^, Holger Hengel^1,2,3^, Idil Cebi^1,2,3^, Johannes Tünnerhoff^3,5^, Maria-Ioanna Stefanou^3,5^, Jonatan F. Hoffmann^3^, Katrin von der Ehe^3^, Johannes Klaus^3^, Julia Vonderschmitt^3^, Matthias L. Herrmann^3,6^, Paula Bombach^3^, Hazar Al Barazi^3^, Lena Zeltner^1,7^, Janina Richter^3^, Klaus Hesse^3^, Kathrin N. Eckstein^3^, Stefan Klingberg^3^, Dirk Wildgruber^3^

# Corresponding author

**Author affiliations:**

1. Department of Neurodegenerative Disease, Hertie-Institute for Clinical Brain Research, and Center for Neurology, University of Tübingen, Germany
2. German Center of Neurodegenerative Diseases (DZNE), Tübingen, Germany
3. Department of Psychiatry and Psychotherapy, University of Tübingen, Germany
4. Department of Epileptology, Hertie-Institute for Clinical Brain Research, and Center for Neurology, University of Tübingen, Germany
5. Department for General Neurology and Stroke, Hertie-Institute for Clinical Brain Research, and Center for Neurology, University of Tübingen, Germany
6. Department of Neurology and Neuroscience, Medical Center, University of Freiburg, Germany
7. Center for rare diseases (ZSE) Tübingen, Germany

**Correspondence to:**

University Hospital Tübingen, Center for Neurology

Dr. Tim W. Rattay

Hoppe-Seyler-Str. 3

72076 Tübingen

Germany

email: tim.rattay@uni-tuebingen.de

phone +49 / 7071 29 82057

fax +49 / 7071 29 4254

## Running title: CSF analysis in patients with psychotic symptoms

**SUPPLEMENTARY MATERIAL**

## Supplementary Table 1: NfL CSF cutoffs

| Age | CSF NfL  upper reference values (pg/ml) |
| --- | --- |
| 20 | 387 |
| 30 | 525 |
| 40 | 713 |
| 50 | 967 |
| 60 | 1313 |
| 70 | 1781 |
| 80 | 2417 |

## Table from Yilmaz^1^ and used for age-dependent cutoffs for CSF NfL measurements.

**Reference:**

**1.** Yilmaz A, Blennow K, Hagberg L, et al. Neurofilament light chain protein as a marker of neuronal injury: review of its use in HIV-1 infection and reference values for HIV-negative controls. *Expert Review of Molecular Diagnostics* 2017;17(8):761-770.

## Supplementary Table 2: Cases with "no reliable classification"

| **ID** | **Diagnosis** | **♀/♂** | **age [years]** | **duration [years]** | **clinical findings / lab results / comments** |
| --- | --- | --- | --- | --- | --- |
| 1 | Schizoaffective disorder | **♀** | 79 | 34 | Long-standing history of schizoaffective disorder. delusions of poverty for >15 years, social isolation, and poor adherence to treatment including medication). CSF revealed marginally elevated neurofilament light chain concentrations of 1782 pg/ml (age-dependent reference <1781 pg/ml) of unknown significance despite further evaluation and examinations. |
| 2 | Funicular myelosis | **♂** | 77 | 1 | Subacute delirious symptoms. Typical MRI abnormalities. Vitamin B12 deficiency with quick clinical improvement after B12 substitution, long-standing history of stomach disturbances as a possible reason for vitamin deficiency. Additional polyneuropathy and optical neuropathy. |
| 14 | Major depressive disorder, recurrent, severe with psychotic symptoms | **♂** | 66 | 24 | Recurrent depressive episodes with psychotic symptoms and pseudodementia. Neurodegenerative markers (CSF) revealed normal tau, p-tau, and ß-amyloid but highly elevated neurofilament light chain concentrations of 1575 pg/ml (age-dependent reference <830 pg/ml) of unknown significance despite further evaluation and examinations. |
| 19 | Reactive major depressive episode with psychotic symptoms, anti-Yo antibodies positive in serum | **♂** | 63 | 0 | Half-year history of rectum adenocarcinoma, which initially received chemotherapy and radiation, then surgical removal (cT3, cN2, G2). Then reactive major depressive episode with acute suicidal thoughts,paranoia (delusions of contamination) and disorganized thinking and speech. Brain MRI without relevant findings or contrast-agent absorbing lesions. Positive anti-Yo antibody in serum which was not examined in CSF. Routine CSF parameters unremarkable. |
| 20 | Persistent delusional disorder | **♂** | 62 | 7 | Dermatozoal delusions without definite proof of a manifest parasitosis, including sporadic subtypes (e.g. Dirofilaria repens). The case remains unresolved with differential diagnoses of delusional disorder or manifest parasitosis. Weak positive ANA-titers (1:320), unremarkable routine CSF parameters, and anti-neuronal antibody screening. |
| 24 | Schizoaffective disorder incl. positive OCBs | **♂** | 56 | 5 | A five-year history of schizoaffective disorder. CSF examination revealed OCBs type II (specific intrathecal IgG production) and a positive MRZ-reaction. Brain MR imaging revealed no relevant increase in white matter lesions with no multiple sclerosis suspect white matter lesions within five years. No autoantibodies were found in CSF or serum, exclusion of infectious CNS diseases. |
| 27 | Schizoaffective disorder incl. MRZ-reaction, OCBs type IV, and positive ANA-titer | **♀** | 55 | 1 | A one-year history of schizoaffective disorder with paranoia (delusion of thought interference and persecution), disturbed thinking and speech, and acoustic hallucinations. Possible autoimmune CSF process with positive MRZ-reaction and OCBs type IV with positive ANA-titer. Unremarkable further routine CSF measurements, anti-neuronal antibody screening and infectious CNS screening. |
| 35 | Paranoid schizophrenia incl. positive intrathecal IgG synthesis, OCBs, and intrathecal CLL cell clones | **♀** | 53 | 2 | Chronic lymphocytic leukemia was diagnosed five years before onset of psychotic symptoms with a watchful waiting strategy. Two-year history of paranoia (delusion of interference, reference, grandeur, surveillance, and posing), thought insertion, and acoustic hallucinations. Partially specific intrathecal IgG synthesis (OCBs type III) with PCR revealed CLL clonal cells intrathecally, which FACS-analysis of CSF did not detect. Brain MRI including contrast-agent not conclusive. |
| 36 | Bipolar disorder with intrathecal measles IgG synthesis | **♂** | 51 | 0 | Long-standing history of bipolar disorder with psychotic symptoms. CSF examination revealed a positive MRZ-reaction and elevated protein. Intrathecal elevated IgG index for measles. Unremarkable further routine CSF findings, anti-neuronal antibody screening, EEG, and brain MRI including contrast-agent. |
| 53 | Paranoid schizophrenia with positive ANA-titer | **♀** | 39 | 6 | A six-year history of psychotic symptoms. Positive serum ANA-titer. Unremarkable routine CSF measurements and anti-neuronal antibody screening in serum and CSF. |
| 66 | Paranoid schizophrenia with OCB type II | **♂** | 32 | 2 | A two-year history of paranoid schizophrenia. CSF examination revealed elevated total protein, IgG index, and OCB type II. Unremarkable anti-neuronal antibody screening in serum and CSF, EEG, and brain MRI. |
| 67 | Paranoid schizophrenia with amygdala lesion | **♂** | 32 | 4 | A four-year history of paranoid schizophrenia. MRI showed T2w increased left amygdala and parahippocampal region signal withno uptake of contrast-agent; this was stable over a four-year follow-up. MR spectroscopy provided no evidence of a brain tumor. Bifrontal rhythmic theta activity in EEG. |
| 71 | Paranoid schizophrenia and neuroborreliosis with improvement under doxycycline | **♂** | 32 | 6 | A six-year history of psychotic symptoms, including disturbed thinking and speech, thought insertion, and acoustic hallucinations. Somatic symptoms, including reoccuring joint pain, muscle pain, paresthesia and hypesthesia, and neuropsychological deficits not typical for paranoid schizophrenia. CSF examination revealed IgM and IgG antibodies against borrelia in CSF and serum. Patient was treated with doxycycline (200mg p.o. daily) for 21 days and improved significantly. |
| 79 | Paranoid schizophrenia with OCB type III | **♂** | 26 | 4 | A four-year history of paranoid schizophrenia. CSF examination revealed OCB Type III. Reduced vision and an episode of hypesthesia in both hands. Brain MRI unremarkable with no uptake of contrast-agent. Unremarkable antineuronal antibody screening in serum and CSF and EEG. |
| 105 | Paranoid schizophrenia incl. positive intrathecal IgG synthesis | **♀** | 20 | 0 | A three-month history of psychotic symptoms with disturbed thinking and speech, delusions of grandeur, and predominant negative symptoms. Possible autoimmune CSF process with OCBs type II (specific intrathecal IgG synthesis). Unremarkable further routine CSF measurements, antineuronal antibody screening, infectious CNS screening, as well as brain MRI. |
| 120 | Paranoid schizophrenia with marginal elevated NfL | **♂** | 27 | 1 | A one-year history of paranoid schizophrenia. CSF examination revealed elevated total protein and marginal elevated NfL with 390 pg/ml (cutoff for age <30 <387pg/ml); compare Supplementary table 1. EEG unremarkable. Brain MRI revealed left-hemispheric white matter lesions; classified as unspecific changes. |
| 123 | Bipolar disorder with hemochromatosis with basal ganglia iron deposition | **♀** | 33 | 0 | Acute onset of manic and psychotic symptoms. Brain MRI revealed bilateral T2w hypointense dentate nuclei, pallidum, and brain-stem nuclei in accordance with brain iron accumulation. Genetic testing with exome analysis revealed homozygous mutations in the HFE gene c.845G>A, p.Cys282Tyr in accordance with porphyria variegate (OMIM ID: 613609). Unremarkable routine CSF parameters and antineuronal antibody screening. |
| 133 | Major depressive episode with psychotic symptoms and marginal elevated NfL | **♂** | 21 | 0 | Acute onset of psychotic symptoms with preexisting major depressive episode. Marginal elevated NfL 395 pg/ml in CSF. Unremarkable further routine CSF measurements, antineuronal antibody screening, EEG, and brain MRI. |
| 136 | Schizophrenia simplex with spike&waves in EEG without epilepsy | **♂** | 25 | 1 | A one-year history of psychotic symptoms. EEG with spike&wave complexes but no clinical epilepsy (neither focal nor generalized seizures). Unremarkable routine CSF measurements, antineuronal antibody screening, and brain MRI. |
| 141 | Schizoaffective disorder incl. serum LGl1-antibodies (1:10) | **♀** | 45 | 0 | Acute onset of psychotic symptoms with paranoia. MRI unremarkable. Treatment with I.v. methylprednisolone and 120mg IVIG (over four days). Initial improvement under concurrant therapy with risperidone. In a two years follow-up with consecutive CSF analysis, LGl1 antibody was negative. No tumor was found despite detailed imaging and clinical examinations. |
| 143 | Paranoid Schizophrenia with OCB type II | **♀** | 32 | 10 | A ten-year history of schizophrenia with one generalized epileptic seizure. Preexisting intellectual disability (IQ 52). CSF examination revealed OCB type II. Unremarkable further routine CSF measurements, antineuronal antibody screening, EEG, and brain MRI. |

Due to the lack of clear diagnostic criteria for F06.2, the cases listed above were not sufficiently explained by abnormal examination results. Therefore, they were recognized as cases with psychotic symptoms and abnormal examination results (n=14 with abnormal CSF and n=7 with normal CSF) and categorized as cases without a reliable classification. The cases are summarized with their abnormal examination findings.
